# Tailoring youth-friendly health services in Nigeria: a mixed-methods analysis of a designathon approach

**DOI:** 10.1080/16549716.2021.1985761

**Published:** 2021-12-14

**Authors:** Ucheoma Nwaozuru, Kadija M. Tahlil, Chisom Obiezu-Umeh, Titilola Gbaja-Biamila, Sarah E. Asuquo, Ifeoma Idigbe, Rhonda BeLue, David Oladele, Kathryn E. Muessig, Nora E. Rosenberg, Jason J. Ong, Adesola Z. Musa, Weiming Tang, Oliver Ezechi, Juliet Iwelunmor, Joseph D. Tucker

**Affiliations:** a College for Public Health and Social Justice, Saint Louis University, Saint, Louis, MO, USA; b Department of Epidemiology, University of North Carolina at Chapel Hill, Chapel Hill, NC, USA; c Nigerian Institute of Medical Research, Lagos, Nigeria; d Department of Health Behavior, University of North Carolina at Chapel Hill, Chapel Hill, NC, USA; e Central Clinical School, Monash University, Melbourne, Australia; f London School of Hygiene and Tropical Medicine, London, UK; g Melbourne Sexual Health Centre, Alfred Health, Melbourne, Australia; h Department of Medicine, University of North Carolina at Chapel Hill, Chapel Hill, NC, USA

## Abstract

**Background:**

Young people in low- and middle-income countries are often neglected in designing youth-friendly health services, especially HIV testing and preventive services. Designathons, which are time-bounded co-creation events where individuals gather in teams to develop solutions to a problem, could promote youth participation and ownership of health services.

**Objective:**

The purpose of this study is to examine youth participation in a designathon to create youth-friendly health services in Nigeria.

**Methods:**

Our designathon was based on crowdsourcing principles and informed by a human-centered design approach. The designathon included an open call for Nigerian youths between 14 and 24 years to share ideas on how to promote uptake of HIV self-testing services and a three-day sprint event that brought together diverse teams to develop strategies enhancing linkage to care. Teams pitched their solutions to a panel of five independent experts who scored ideas based on the desirability, feasibility, potential impact, and teamwork. We used descriptive statistics to summarize participants’ demographics and conducted a content analysis to synthesize themes from youth proposals.

**Results:**

Nine hundred seventy-six youth across Nigeria applied to join the designathon. Forty-eight youth in 13 teams participated in the designathon with a median age of 20 years (IQR: 17–22]. Boys and young men were 48.5% (446/919) of the total applicants, 62.5% (30/48) of the designathon participants, and 63.6% (7/11) of the finalists. Students, from all educational levels, represented 91.2% (841/922) of the total applicants, 88.4% (38/43) of the designathon participants, and 90.0% (9/10) of the finalists. About twenty-three percent (3/13) of the final proposals were top ranked. The three finalist approaches to optimize youth-friendly health services centered on decentralizing service delivery to young people through mobile health technologies, use of mobile tents, or peer support services.

**Conclusions:**

Our open call engaged diverse groups of Nigerian youth, including young women and students. Our data suggest that designathons may be useful for developing tailored youth-friendly health services. Further research is needed to understand the designathon process and the effectiveness of the finalist submissions.

## Background

Youth-friendly health services are increasingly recognized as essential to promote HIV testing, linkage to care, treatment initiation, and retention among youth (14–24 years old) [1]. The World Health Organization (WHO) defined youth-friendly health services as services that are accessible, acceptable, equitable, and appropriate to youth [2,3]. Barriers to youth-friendly HIV services include concerns about confidentiality, few youth-friendly clinics, and health systems focused on adults [4,5] The WHO has recommended youth-friendly health services to improve outcomes, tailor services, and better meet the unique needs of youth.

In line with the WHO priorities to make health services more youth-friendly, Nigeria launched the National Strategic Framework on the Health & Development of

Adolescents & Young People in Nigeria in 2007 [6]. Despite high-level support for youth-friendly health services in Nigeria, there has been little progress to achieve these goals. For example, Nigerian youth have low rates of HIV testing and usage of HIV prevention services. Only about 25% of Nigerian youth aged 14–24 years have ever tested for HIV [7], and they are the least likely age group to be linked to care promptly following a positive HIV test result [8,9]. The low uptake of testing services and poor rates of linkage to care can partly be attributed to the lack of youth-friendly services [10,11]. Provision of HIV testing options such as HIV self-testing, which is a process that allows individuals to collect their blood or oral fluid sample, conduct the diagnostic test, and then interpret their results [12], within the context of youth-friendly health services may promote HIV testing among young people.

Despite the importance of developing youth-friendly health services, optimal methods for tailoring health services for youth are not well understood [10,11]. Some strategies for tailoring health services have been developed by adults, with minimal or tokenistic input from youth themselves [13]. Strategies that meaningfully engage youth to develop and establish effective modalities for delivering youth-friendly health services are needed. Participatory approaches such as designathons could increase youth ownership of these health services by providing opportunities for young people to solve health problems that affect them. Designathons are intensive, time-bounded, co-creation events where individuals work in teams to develop solutions to a problem [14]. This approach can foster multi-disciplinary collaborations and has generated innovative solutions in education, technology, and public health [14,15].

To address the gap in youth engagement in developing youth-friendly health services, we organized a designathon in partnership with 4YouthByYouth (4YBY) [16]. The 4YBY is a team of young people, health professionals, activists, and entrepreneurs from diverse backgrounds, who are united by their shared passion to advance Nigerian youth participation in creating innovative, sustainable HIV services [16]. The designathon used a human-centered design framework, an approach that involves end-users to develop a deep understanding of their needs and to maximize user satisfaction [17]. The objective of this paper is to describe the designathon and its participants, summarize team proposals, and discuss implications for youth-friendly health services in Nigeria.

## Methods

**Figure 1.**
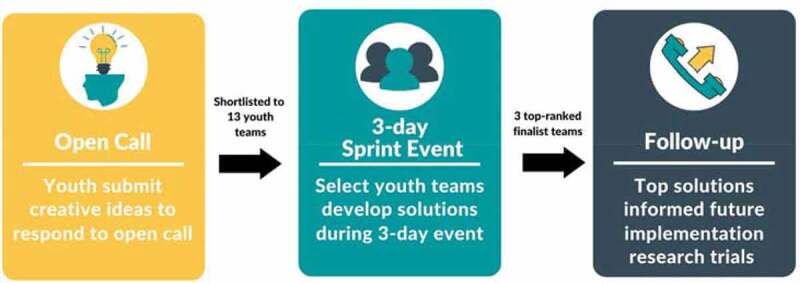
4YBY designathon open call process

Note: The three-phased process included an open call for teams of young people to submit their ideas on how to design youth-friendly health services. The top 13 ideas submitted were shortlisted to participate in a 3-day sprint event after three ideas emerged as top solutions to design youth-friendly health services and will inform future implementation research trials by the research team.

We combined a crowdsourcing approach with human-centered design principles to conduct a designathon- open call implemented in three phases: an open call, a three-day design sprint event, and a follow-up phase (Figure 1). The crowdsourcing open call followed the stages recommended by the WHO/TDR practical guide [18]. The sprint event, held from March 20th to 22nd 2020 in Lagos, was dedicated to developing new HIV self-testing delivery services and devising linkage strategies to youth-friendly health services. The 3-day sprint event occurred immediately prior to and during an official nationwide lockdown, due to the COVID-19 pandemic that took effect on March 22^nd,^ 2020.

### Phase I: Open call

We convened an open call advisory committee comprised of six individuals who were representatives of partnering organizations, young people, youth groups, public health institutions, and state ministries of health. The open call advisory committee guided the overall development, promotion, and evaluation of the open call. Individuals could submit ideas to the open call online (i.e. via Google online forms, WhatsApp, or email) or offline (i.e. paper-based forms). Paper forms were accepted in Lagos at the Nigerian Institute of Medical Research. Participants interested in joining the open call completed an online informed consent. The open call was disseminated on social media platforms, secondary schools, university campuses, and community centers where young people congregate. The open call was also promoted on the 4YBY website (Supplement 1) and social media (Twitter, Facebook, Instagram) [19].

The open call prompts were developed through an iterative process. The authors created an initial draft of the prompts and solicited feedback on the wording and design of the prompt from the open call advisory board which consisted of young people and community leaders in Nigeria. To enhance youth engagement, we created three specific prompts: 1) How might we deliver HIV self-testing services and other youth-friendly health programs to young people in ways that are low-cost, acceptable, appealing, and confidential? 2) How might we link young people to HIV self-testing and other youth-friendly health services that are low-cost, acceptable, appealing, and confidential? 3) How might we make youth-friendly health services a place that young people would want to go for STI testing? Participants submitted their responses to one of the three open call prompts, in the form of written descriptions (150 words or less), images, drawings, posters, videos, and taglines. To be eligible to participate in the open call, participants were required to provide a signed consent form and acknowledge they were between the ages of 14–24 years as of the open call start date and reside in any state in Nigeria.

### Phase II: Sprint event

The submitted entries were internally vetted by the research team members to generate a refined list of top entries, excluding ideas that were not feasible, duplicate entries, or irrelevant. Following the idea vetting process, the quality of ideas was scored by the open call advisory committee based on three criteria: desirability to young people, feasibility to implement, and potential for impact. Each criterion was evaluated on a 10-point scale, with one being the lowest possible score and ten being the highest. Entries within the top 40 percentile were invited to the three-day, in-person sprint event. This sprint event provided an opportunity to refine and develop a prototype of the proposed youth-friendly service. The designathon participants received one-on-one mentorship from industry experts, with common interests in youth entrepreneurship and health, to guide them through creating prototypes of their services, conducting research, and mapping out the user journey. Subsequently, participants presented their ideas to an expert panel of judges in a 5-minute pitch presentation. Judges assessed the team’s deliverables (i.e. proposal pitch and user journey map) based on feasibility, desirability, impact, and teamwork. Judges included local and national key stakeholders such as community youth representatives, public health professionals, entrepreneurs, and marketing professionals in Nigeria. Following the judging process, all teams were acknowledged in the form of participation certificates and the first three places were recognized with prizes.

In an effort to mitigate the spread of the novel coronavirus, COVID-19, and in adherence to the infection control guideline of the Nigeria Centre for Disease Control (NCDC), we implemented several measures to minimize person-to-person contact during the three-day sprint event. Some of the measures included practicing social distancing (at least 3 meters apart), handwashing, routine cleaning of frequently touched surfaces, and limiting attendance and seating capacity to 50 persons or less. Following the event, the research team conducted a daily symptom check for fourteen days among all participants who attended the event. The symptom check was based on self-report by the designathon participants.

### Data analysis

We performed descriptive statistics to characterize demographic information of designathon participants including their age, gender, the highest level of education completed, and occupation. All analyses were performed using SAS version 9.4 (SAS Institute). We also conducted a content analysis, which provides a practical guide to illuminate new insights and knowledge-based replicable and valid inferences from data [20,21]. A deductive approach to content analysis was chosen given that the human-centered design thinking framework guided the design and analysis process of the designathon. Deductive content analysis has been used in previous studies to provide a guiding frame and a structure for categorizing qualitative data and study findings [22,23]. The deductive content analysis involved three main phases: (1) preparation phase relates to the selection of the unit analysis [24]; (2) organizing phase involves open coding, generating and grouping similar topics together to form categories; (3) reporting phase relates to generating a model or conceptual map to present the results. The units of analysis were the User Journey Maps and proposal pitch generated from each of the thirteen teams who participated in the sprint event. Journey maps offers pictorial illustrations of user experience through accessing and interacting with a service or product over time and have been used by researchers and practitioners for supporting the analysis and design of blueprints for new health services [25–27]. The journey map of the top-ranked finalist designathon team is included as a supplement (Supplement 2). The responses to the journey map as well as the proposal pitch were consolidated to create a single transcript for each team which resulted in 13 discrete transcripts. To create an initial coding matrix, two researchers independently coded four transcripts. A coding meeting was held where both researchers discussed discrepancies and differences in coding to reach a consensus. Following the creation of the coding matrix, a content analysis of all transcripts was performed by the two researchers.

### Ethical approval

Ethical approval to conduct the research was received from the Nigerian Institute of Medical Research Institutional Review Board, the Saint Louis University Institutional Review Board, and the University of North Carolina Institutional Review Board.

## Results

### Participant demographics

Nine hundred and seventy-six Nigerian youth in 248 teams applied to participate in the designathon, of which 48 were invited to join in the designathon in 13 teams (Table 1). Eleven youth in three teams were selected as designathon finalists. The median age of designathon participants was 20 years (interquartile range 17 to 22 years), which was five years higher than the median age of the designathon applicant pool and three years higher than the selected designathon finalists. The majority of designathon participants (62.5%) and finalists (63.6%) were male, contrasting the majority of female designathon applicants (51.5%). Although 14.5% of designathon applicants reported their highest level of education as primary school, all designathon participants and finalists had at least a secondary level of education. The majority of designathon participants (88.4%) and finalists (90.0%) were students, which was consistent with the majority student designathon applicant pool (91.2%). The designathon participants resided in various states, the majority (38.5%) resided in Lagos State, followed by (23.1%) in Oyo, (15.4%) in Ondo, and (7.7%) in Abuja, Benue, and Enugu, respectively.

**Table 1.** Socio-demographic characteristics of designathon applicants and participants: Nigeria 2020

	**Designathon Applicants** **(N = 976)**	**Designathon Applicants from Top 40 Teams** **(N = 157)**	**Designathon Participants** **(N = 48)**
	**n (%)**	**n (%)**	**n (%)**
**Age (years)**			
Median (Interquartile range)	15 (14–18)	17 (15–22)	20 (17–22)
**Sex**			
Female	473 (51.5)	60 (41.1)	18 (37.5)
Male	446 (48.5)	86 (58.9)	30 (62.5)
Missing	57	11	0
Highest Level of Education*			
Primary	132 (14.5)	21 (14.6)	0 (0)
Secondary	633 (69.3)	70 (48.6)	20 (45.5)
Some Tertiary	44 (4.8)	19 (13.2)	13 (29.6)
Tertiary	104 (11.4)	34 (23.6)	11 (25)
Missing	63	13	4
**Occupation**			
Employed, Self Employed or NYSC^†^	58 (6.3)	17 (11.9)	4 (9.3)
Unemployed	23 (2.5)	6 (4.2)	1 (2.3)
Student	841 (91.2)	120 (83.9)	38 (88.4)
Missing	54	14	5

*In Nigeria, primary education is elementary schooling for six years, which is then followed by secondary schooling for six years. Tertiary education is post-secondary schooling in which students attend universities, polytechnics, monotechnics, or colleges of education.

†NYSC = National Youth Service Corps

### Youth-friendly health services

The three finalist designathon teams proposed potentially feasible, desirable, and impactful strategies to deliver youth-friendly health services, as determined by the judges (Table 2).

**Table 2.** Proposed strategies to develop youth-friendly health services by the three finalist teams at the designathon: Nigeria 2020

Rank	Team Name	Team Members*	Team Location (State)	Team Size	Youth-Friendly Health Services Proposals	Total Score^†^
1	iDoc	Tertiary Students	Oyo	3	Develop a mobile-friendly web app for youth to access sexual and reproductive health services. Use emerging technologies, such as internet-of-things, to deliver health information between youth and health providers and initiate linkage to care. Youth can order HIV self-test kits with attached sensors through the app, which will monitor when test kits are open and notify health providers.	158
2	SafeTent	Tertiary Students NYSC Members	Lagos	3	Launch mobile and stationary tents, consisting of basic medical services and fun activities, to recruit youth and deliver health services. Youth who visit these tents can purchase a bag containing various health products. The study team will follow-up, through phone calls or email, to see if youth who visited the tents utilized any of the health products.	142
3	Edoro	Secondary Students	Lagos	5	Establish multisectoral collaborations to promote and deliver health services. The study team will provide youth with health kits at schools or community health fairs. The study team will also offer peer support services to encourage the use of health kits and linkage to care.	139

NYSC = National Youth Service Corps

*In Nigeria, primary education is elementary schooling for six years, which is then followed by secondary schooling for six years. Tertiary education is post-secondary schooling in which students attend universities, polytechnics, monotechnics, or colleges of education.

†Range of possible total scores was 0 to 200 points. Total score was determined by five judges who each evaluated the designathon proposals on potential desirability, impact, feasibility and teamwork. Each criterion was on a 10-point scale.

The top-ranked finalist team comprised of an all-male team of three tertiary students. Their proposed idea entailed the development of a mobile phone application that would allow youth to identify nearby youth-friendly health services. Youth could then connect to health providers using Internet-of-Things technology, a method in which multiple devices communicate with each other, using the internet, to send or obtain information. Youth will be able to order HIV self-test kits with attached sensors through the mobile phone application. When youth open the test kits, providers from a health center identified and selected by the youth will receive a notification. This notification will prompt providers to reach out to youth and link them to care. The team’s use of the Internet-of-Things is an example of utilizing emerging technologies to deliver youth-friendly health services.

The second-ranked finalist team consisted of an all-female team of two tertiary students and one member of the National Youth Services Corps, a one-year mandatory national service program for individuals who have completed their university studies. Their proposed strategy was to create mobile and stationary tents in communities to deliver youth-friendly health services. Youth who purchase health products during their visits in the tents will be followed up with by the study team to ensure the utilization of the health products and link them to care. The team’s use of mobile and stationary tents is an example of a community-based model in which the intervention is delivered in communities, increasing accessibility for youth.

The third-ranked finalist team included four males and one female youth in secondary school. Their proposed idea was to deliver youth-friendly health services through schools and community health fairs. The team will provide kits containing health products to youth and collect their health information. They will also create peer support services through trained peer counselors to encourage youth who received health kits to utilize the products, adopt healthy behaviors and link them to care. The team also described their intent to establish collaborations with different governmental agencies and educational institutions to help promote and deliver health services. The team’s plan to partner with governmental and educational groups can help make community buy-in successful, can ensure coordination of health services, and can help the recruitment of youth for the intervention.

The other ten teams at the designathon also proposed potentially feasible strategies to deliver youth-friendly services to youth (Supplement 3). One team proposed setting up an Unstructured Supplementary Service Data (USSD) code or a toll-free call center where youth can connect with representatives from a youth-friendly health center and receive health services. Four teams planned to provide youth-friendly health services through their mobile application or website, giving youth access to important resources. Two teams proposed the creation of a youth hub where young people can regularly congregate to socialize and receive health services. One team planned to conduct public health awareness campaigns to encourage youth to attend a nearby youth-friendly health center to receive care. One team proposed creating and distributing kits with multiple sexual and reproductive health products and sending enhanced reminders for follow-up. One team planned to conduct youth workshops in secondary schools, teaching students about available health resources in their communities.

Among the 13 designathon teams, we identified common themes across their proposals. One theme was the proposed development of mHealth interventions to increase uptake of health services. Teams recognized the expanding use of mobile phones among youth and the opportunity for youth to seek health services through mobile devices. Another theme was the provision of peer support to encourage the adoption of healthy behaviors. Youth teams at the designathon recognized the influence of peer support as a potential HIV prevention strategy. A third theme was the utilization of different institutions and media outlets to promote youth-friendly health services. Teams recognized that multi-sectoral collaborations allow stakeholders to be included in the development and sustainability of youth-friendly health services.

### Follow-up activities

Following the completion of the sprint event, the ideas generated informed the refinement of a youth-friendly HIV self-testing intervention delivery to be evaluated in an RCT (ClinicalTrials.gov **Identifier**: NCT04710784). In addition, two of the secondary schools that participated in the designathon open call expressed interest in implementing youth-led health clubs within their institutions to increase knowledge and awareness about HIV and HIV self-testing.

## Discussion

Our designathon provided a rich set of participatory activities to facilitate meaningful youth engagement, helping to tailor youth-friendly health services. Forty-eight young people across Nigeria, comprised of thirteen unique teams, generated diverse and creative strategies to enhance youth-friendly health services. The designathon was useful in generating innovative ideas and contributes to the limited literature on the use of crowdsourcing approaches for health innovation for young people in sub-Saharan Africa [16,28,29]. We extend the literature by focusing the designathon on tailoring of youth health services for Nigerian youth and the implementation of the sprint event during the COVID-19 pandemic social gathering restrictions.

We found that the youth teams were able to develop solutions that were highly ranked among independent external reviewers. These findings are consistent with previous designathons where high-quality proposals and ideas emerged at the end of the event [30]. However, designathons and similar events may not have ideas translated into programs or research studies. Our research team will be working with the finalist teams to incorporate elements of their solutions as part of a pragmatic clinical trial. Specifically, these elements would constitute the intervention that seeks to promote the uptake of HIV self-testing and linkage to youth-friendly health services for HIV care and preventive services among Nigerian youth.

Our findings also show high participation and engagement among students. Over 90% of the designathon open call entries and finalists were students in either universities or secondary schools. Students from a wide range of academic backgrounds, age groups, and disciplines played a pivotal role in the designathon activities. This is consistent with other designathon literature that has successfully engaged a diverse group of students to create innovative health solutions [30,31]. This finding also suggests that schools and universities may be a key partner for institutionalizing designathons within some communities. The creation of school-based clubs could help to sustain this approach over time and build capacity for subsequent youth-friendly service projects. Schools and universities may be a good platform to organize designathons to tailor health services.

We observed a large number of boys and men as designathon applicants, participants, and finalists. Although the designathon ideas were not gender-specific the high percentage of boys and young men (48.5%) in the development of HIV services using this participatory approach is noteworthy. While previous literature shows low male involvement in the delivery and uptake of HIV preventive and care services [32–34], this designathon underscores the capabilities of young men to develop promising proposals for youth-friendly health services. This participatory approach is comparable to other open challenge contests that have been successful in engaging men in developing interventions to promote health care utilization [35]. Designathons may provide an enabling environment for male participation in the development of youth-friendly health services.

Our data demonstrate that designathons can be implemented with COVID-19 precaution measures in place. This is consistent with other hackathons that were successfully implemented during the COVID-19 pandemic [36,37]. In contrast to our designathon, these events were conducted virtually [36,37]. Our designathon was implemented immediately prior to the COVID-19 outbreak in Nigeria. Enactment of measures to prevent the spread of COVID-19 in Nigeria included the ban on gatherings of more than 50 people. In accordance with directives, the designathon was executed utilizing multiple halls at the Nigerian Institute of Medical Research (NIMR) to ensure that less than 20 individuals were present in each room at all times. This process ultimately provides evidence on the feasibility of in-person designathons during restrictive social gathering scenarios presented by the COVID-19 pandemic. Given that the designathon involves a small number of individuals and the availability of the spaces at NIMR, it was possible for the designathon components to be achieved with minimal modifications to ensure the safety of the participants and staff. However, it is important to note that it would not be possible to execute a designathon during a total lockdown, given the restriction on any form of in-person gatherings.

Our process has some limitations. First, the findings reported here are based on the activities and outcomes of this specific designathon, and although we believe that features and lessons learned may be useful for other similar events, readers need to consider the context of this open call in assessing the extent of its transferability to different situations [38]. Second, there was no longitudinal follow up performed among the designathon participants, beyond the top three teams. Therefore, we cannot ascertain if the solutions proposed by the teams were tested or developed beyond the designathon period. In terms of research, a more rigorous evaluation of ideas created in designathons is needed. Third, participants for the designathon were recruited through promotions online, and in-person announcements and posters in schools and community-centers. Therefore, our reach is limited to young people who have access to these locations. For future designathons, we will be making concerted efforts to utilize recruitment strategies that allow us to reach out young people in non-formal settings and in more rural settings. In addition, it would be important to consider youth with disabilities and other marginalized subsets of youth. More community engagement with these subsets of youth (and their inclusion on steering committees, judging groups) could help in future open calls to make this process more inclusive. Finally, the designathon is capital and time-intensive, which can pose a challenge in resource-limited settings. To minimize costs, we leveraged local resources, such as the Nigerian implementing team comprising of staff from the Nigerian Institute of Medical Research, and volunteers [30].

### Implications

This designathon has important implications for public health research and policy. First, evidence from this process can inform the active engagement of young people to generate solutions to issues that affect them. Particularly, the potential of academic institutions as a hub to foster youth co-creation and innovation in all sectors in Nigeria, beyond health. Second, the designathon offers insights on the role of stakeholder engagement in the successful implementation of the designathon, even in unconventional contexts such as social gathering restrictions with the COVID-19 pandemic. Third, it can inform the creation of sustainable youth-friendly service models in Nigeria, as the proposed solutions are developed by young people who are the end-users of the service. The findings further suggest the need for longitudinal follow-up after the designathon to evaluate the impact and sustainability of proposed interventions. In addition, further research is needed to evaluate the processes and outcomes of the designathons, and to optimize the methods for implementation science, which seeks to increase the translation of evidence-based interventions to real-world settings.

## Conclusions

Against the backdrop of promoting linkage to care and preventive services among young people through the creation of youth-friendly health services, a designathon provided the necessary catalyst to spur innovative solutions among Nigerian youth. This designathon provides evidence to shape the delivery and implementation of youth-friendly health services among Nigerian youth. Despite the successes of the 4YBY designathons [30], designathons in Nigeria is a nascent concept, and there remains substantial room for innovation and improvement to foster the translation of ideas generated to reality. To this end, the 4YBY research team will be incorporating components of the solutions generated from the designathon in a randomized controlled trial that seeks to promote the uptake of HIV self-testing and linkage to youth-friendly health services for HIV care and preventive services among Nigerian youth. Findings from this study will expand the body of work on the continuity and sustainability of solutions generated from time-bounded co-creation events, such as designathons.

## Supplementary Material

Supplemental Material

## Abbreviations

4YBY: The 4 Youth By Youth

HIV: Human Immunodeficiency Virus

IQR: Interquartile Range

NYSC: National Youth Service Corps

STI: Sexually transmitted infections

USSD: Unstructured Supplementary Service Data

## Paper context

Young people in low- and middle-income countries including Nigeria are often neglected in designing youth-friendly health services, especially HIV testing and preventive services. Participatory approaches such crowdsourcing has the potential to address this gap by engaging young people to co-create strategies to increase the uptake of youth-friendly health services.
