# Supplementary material for: Tailoring youth-friendly health services in Nigeria: a mixed-methods analysis of a designathon approach
Source: Glob Health Action. 2021 Dec 14;14(1):1985761. doi: 10.1080/16549716.2021.1985761 (PMC8676684; doi:10.1080/16549716.2021.1985761)
Supplement: Supplemental Material [file ZGHA_A_1985761_SM2618.docx]

SUPPLEMENT 1. The 4YouthByYouth open call for submissions


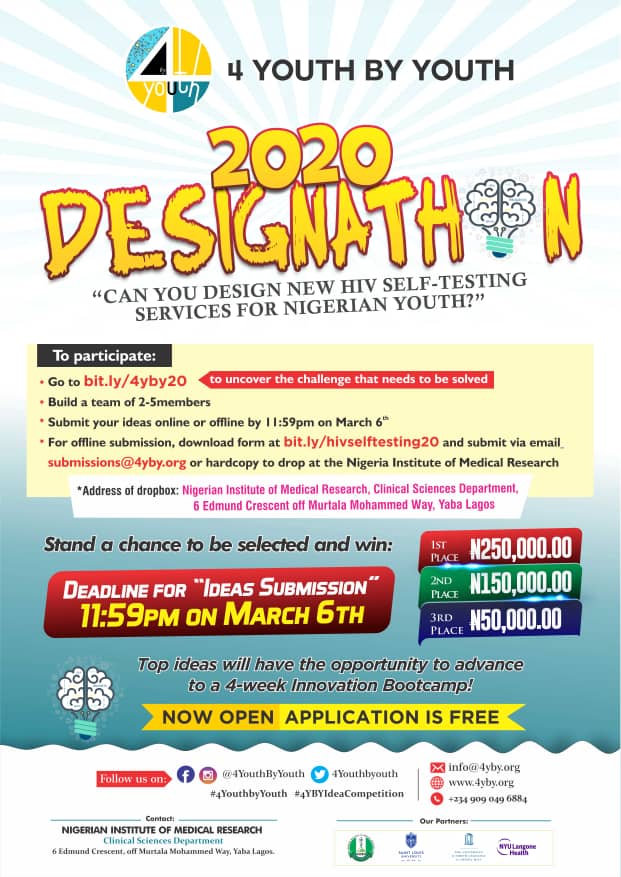


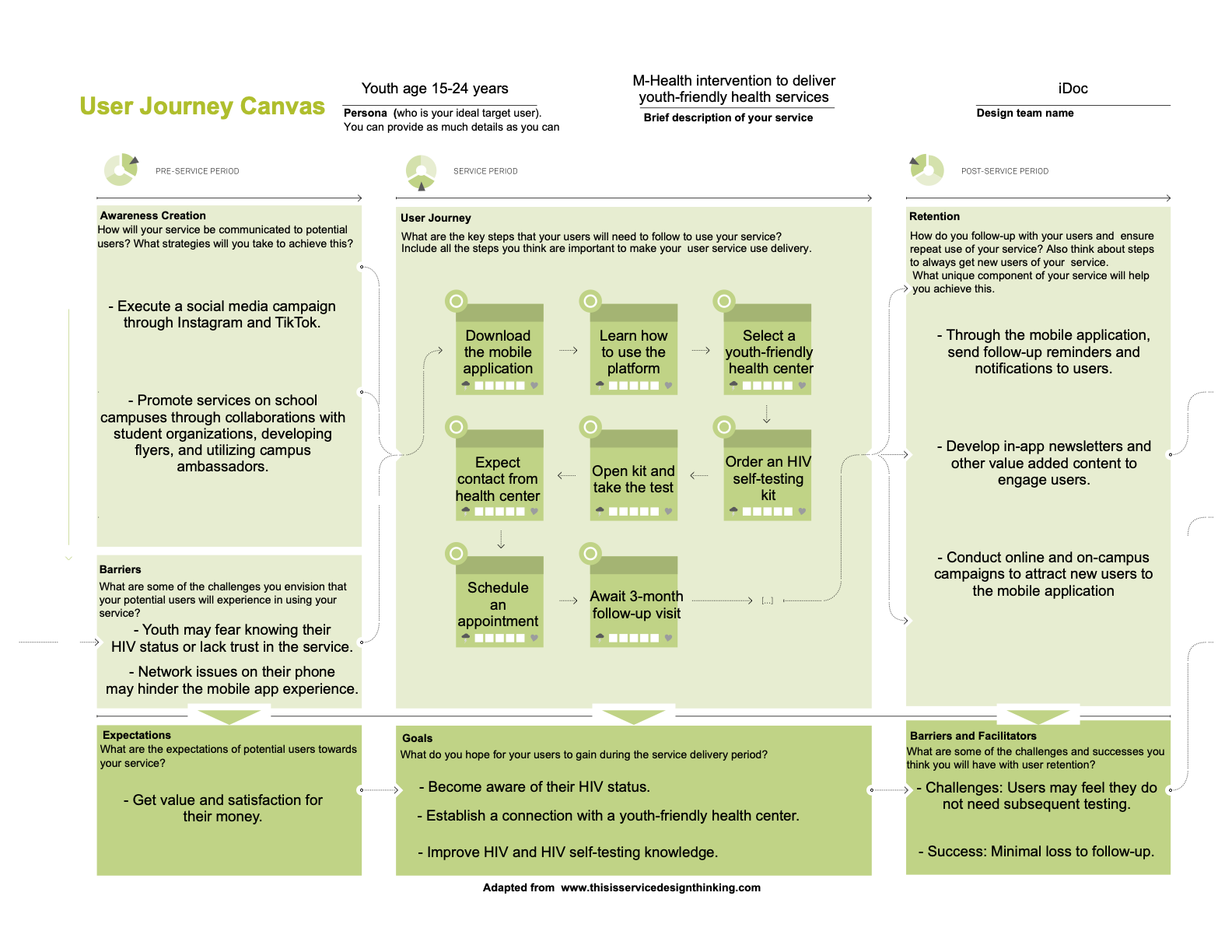
SUPPLEMENT 2. User journey from the top-ranked finalist team

| SUPPLEMENT 2. Proposed strategies to develop youth-friendly health services by teams ranked 4th through 13th at the designathon: Nigeria 2020 | | | | | | |
| --- | --- | --- | --- | --- | --- | --- |
| **Rank** | **Team Name** | **Team Members*** | **Team Location (State)** | **Team Size** | **Youth-Friendly Health Services Proposals** | **Total Score†** |
| 4 | Crilsta | Tertiary Students | Oyo | 5 | Create an e-commerce website which youth can use to access health services. Kits containing selected health products can be delivered to the youth's preferred location or can be picked up at a nearby youth-friendly health center. The study team will follow-up with youth through email, SMS or phone calls. | 136 |
| 5 | Leverage | Tertiary Students | Oyo | 2 | Create a kit containing sexual and reproductive health products (i.e., condoms and sanitary pads) and a user pamphlet. The study team will conduct a one-week follow-up, send enhanced reminders, introduce youth to peer-support groups, and refer youth to a health centers. | 132 |
| 6-Tie | Marigold | Secondary students | Lagos | 3 | Conduct training workshops at secondary schools to teach students about available health services. Students will learn about resources in their communities and receive counselling. | 125 |
| 6-Tie | Age | Tertiary Students | Enugu | 5 | Hold public health awareness and advocacy campaigns and deliver health services at locations frequented by the tended youth population. Youth can call a USSD/toll-free call center to receive counselling services and be linked to a nearby youth-friendly health center. Follow-up will be conducted by volunteers and youth-friendly health service workers. | 125 |
| 8 | Segura | Tertiary Students | Benue | 2 | Conduct multi-channel health awareness campaigns to encourage youth to attend a nearby youth-friendly health center where they can receive health services. The study team will conduct follow-up phone calls. | 117 |
| 9-Tie | DevTrain | NYSC Members | Abuja | 5 | Trained peer educators will recruit youth to join a hub where they can receive sexual and reproductive health and counseling services. Youth will also be able to learn about available health resources from the hub's website. The study team will then follow up with youth who utilized services to triage them to a nearby health center. | 114 |
| 9-Tie | Didi Godswill | Employed and Unemployed Members | Ondo | 3 | Create a kit containing sexual and reproductive health products. The kits will be marketed online and offline, and sold in various locations. Youth who purchase the kits will be required to provide contact information so the study team can follow up and refer them to peer support groups and youth-friendly health centers. | 114 |
| 11 | Exceptional | Tertiary Students | Ondo | 4 | Develop a web app that will allow youth to access health services. Youth who sign up on the app and receive services will be contacted through SMS, emails or phone calls for follow-up. Youth will also be able to find a nearby youth-friendly center through the app. | 105 |
| 12 | United Stars | Secondary students | Lagos | 5 | Form youth health clubs where youth will regularly meet and be able to receive health kits, learn more about available health resources, and develop a peer social network. | 102 |
| 13 | X3 | Tertiary Students | Lagos | 3 | Develop a web app that will allow youth to access health services and find nearby youth-friendly centers. The study team will send follow up reminders through the app or email. | 102 |
| NYSC = National Youth Service Corps; USSD: Unstructured Supplementary Service Data *In Nigeria, primary education is elementary schooling for six years, which is then followed by secondary schooling for six years. Tertiary education is post-secondary schooling in which students attend universities, polytechnics, monotechnic, or colleges of education.  †Range of possible total scores was 0 to 200 points. Total score was determined by five judges who evaluated the designathon proposals on potential desirability, impact, feasibility and teamwork. Each criterion was on a 10-point scale. | | | | | | |
